# Beyond Individual Illness: Using Illness Narratives to Explore the Navigation of *Care* and *Work* When a Relative Is Dying

**DOI:** 10.1111/1467-9566.70188

**Published:** 2026-04-28

**Authors:** Kate Reed, Laura Towers

**Affiliations:** ^1^ Sociology The University of Manchester Manchester UK

## Abstract

Sociologists have frequently written about family involvement in end‐of‐life care, highlighting the emotional and physical labour involved. Research has also examined how individuals juggle paid work commitments with informal long‐term care. Less is known about the impact of illness trajectory on care and work in the temporarily ambiguous context of terminal illness. Drawing on qualitative data collected using in‐depth interviews and analysed thematically, this article explores how individuals manage paid work commitments when they are informally caring for a relative who is terminally ill. Informed by illness narratives as retold by bereaved relatives, this article examines the impact that illness can have on a ‘carer’s’ ability to engage in paid work. This article concludes by highlighting the temporal and socially embedded nature of care. By uncoupling illness experience from the individual sufferer and using it as a vehicle through which to analyse other social *lives* and social *phenomena* (namely, work and care), this article offers a novel contribution to the sociology of health and illness and to wider sociological discussions on the use of narrative. It also extends the focus of sociological debates on care by problematising the relationship between care and work across the boundaries of life and death.

## 1 Introduction

Terminal illness is a frightening and uncertain experience for individuals and their families (McNamara 2001). Past, present and future conceptualisations of time become ambiguous in the face of end‐of‐life uncertainty (Ehlers et al. 2024; Rovers et al. 2019). Individuals who are terminally ill often spend significant amounts of time in hospital or in a hospice. Scholars have frequently shown that a ‘good death’ is more likely to occur when people die in a place of their preference (Krikorian et al. 2020), which, for most people, is at home (McNamara 2001). Sociologists have explored the role that family often plays in providing care in this situation, illuminating the physically demanding and emotional nature of such care (Wilkinson and Wilkinson 2020). Less is known about how individuals manage the relationship between care and work in the uncertain terrain of terminal illness, nor is it known how the nature and meaning of care can change across the boundaries of life and death.

Research has frequently shown how those involved in the long‐term care of family members (e.g., childcare or care of an ill or infirm family member) do not see paid employment as compatible with their care responsibilities (Barrett et al. 2014). Those involved in care often retire early or reduce their working hours to fulfil various caregiving obligations (Carmichael and Charles 2003; Geyer 2016). Existing research on work and care can include a focus on the effects of potentially temporally specific forms of care, including care of a relative with a life‐threatening illness (Bijnsdorp et al. 2021). The concept of care in such research, however, is often treated uncritically and fails to distinguish between care as a *feeling* or *activity state* (Thomas 1993, 652). Drawing on qualitative research conducted with the Compassionate Employers team at Hospice UK, we aim to scrutinise the relationship between care, work and terminal illness. Care in this context, we argue, is diverse and depends on the type of social relationship involved, as well as the form and progression of illness. By illuminating how people manage their professional lives while caring for a terminally ill relative, this article extends the focus of existing sociological research on work and care. It also contributes new knowledge to sociological understandings of the concept of care in the specific context of terminal illness.

Research on end‐of‐life *care* has tended to be situated in the field of death studies—an umbrella term for research spanning all aspects of death, dying and bereavement (Borgstrom and Ellis 2017). Scholars of death and dying have increasingly sought to analyse individual experiences of the dying process, especially those documented through online public accounts (Brennan 2024). This article is informed by this experiential focus but takes the narrative of illness approach as its inspiration and is situated explicitly within the sociology of health and illness. The rationale behind this positioning lies in our specific focus on the social impact of illness in this context. As Riessman (2015) has argued, illness narratives are socially embedded and co‐constructed, infused with inter‐relationality and a consciousness of others. Illness narratives often act as a reference point between the individual and society (G. Williams 1984), uncovering the dynamic relations between people and their social contexts (Good 1994, 161). They also enable individuals to reconstruct or project identity in the event of illness (Hydén 1997, 155). Rather than using narratives of the ill person, however, this article draws on illness stories as retold by bereaved relatives to explore the impact of terminal illness on their ability to juggle informal care activities and paid work. Through this process, we will unpack the nature of care in this context, highlighting the importance of medically focused tasks. By using illness narratives as a reference point through which to examine the relationship between *other* individuals (beyond the illness sufferer) and *their* social worlds, this article seeks to offer a unique contribution to the sociology of health and illness and to broader sociological discussions on narratives.

In this article, we will also explore the temporally disruptive nature of terminal illness, as well as examining structural and organisational impediments to combining care and work. This article will conclude by illuminating the ways in which the *nature* and *meaning* of care change as death approaches, with care as a *feeling* state taking priority over care as an *activity* state (Thomas 1993). In doing so, we seek to encourage sociologists to rethink the ways that care is conceptualised across life and death and in different settings. We begin in the initial section by outlining background literature along with the project's conceptual focus and method before moving on to a discussion of the study findings presented in three sections: illness narratives and the meaning of care; managing illness, managing work and ‘time to care’; and identity, work and disease progression.

### 1.1 Conceptualising Care *and* Work

Care is a relational activity centred around meeting needs (Tronto 2015). It is structured through time and shaped by social, economic and political structures (Peterie and Broom 2024; Ihlebæk 2021). Families play a crucial role in providing care for their members at different points across the life course (Barrett et al. 2014). Such commitments frequently compete with other life stage responsibilities as well as the pressures of paid employment (Yeandle et al. 2002). For example, international research has shown how those involved in long‐term care often retire early, reduce work hours, take on fewer responsibilities or forego promotion to fulfil caregiving obligations (Carmichael and Charles 2003; Geyer 2016). Nonwork factors such as dealing with difficult family members can also complicate the balance between work and care (Gaugler et al. 2018). Structural problems, such as workload, unsupportive colleagues and line managers, can create further obstacles. As Geisen et al. (2024) argue, the challenges of combining care and work often become more visible when new or changing care needs related to providing care disrupt established work–life patterns.

Although caring responsibilities often clash with paid employment commitments, care is also often framed as a form of work (Graham 1983). Care is a time‐consuming and often gendered activity (Davies 1994; Graham 1983) involving both emotional and physical labour (Carr and Biggs 2018; Herron et al. 2019; Reed and Ellis 2020; Reed et al. 2023; Thomas et al. 2002). It is, therefore, often treated as a form of dirty work, a hidden and frequently devalued form of labour (Davies 1994; Twigg 2000). In recent decades, there have been attempts within UK health policy to positively reframe a carer's identity to that of an ‘expert carer’ through training provision. Such initiatives are part of a wider health policy shift focused on empowering patients and their supporters to become lay health experts able to self‐manage illness (Department of Health 2008). Sociologists have often been critical of such training schemes. For example, Sadler et al. (2018) found the process of training informal carers in stroke units was not simply a case of skill transference but constituted a form of disciplinary power intended to shape the conduct of the carer.

Although care *as* a form of work, and care *and* paid work, has been given some attention in sociology, research has tended to focus mostly on long‐term care. Less is known about the challenge of combining care of a terminally ill relative with paid work commitments. As end‐of‐life scholars have highlighted, informal care of a dying relative is often fraught with emotional, financial and environmental challenges (Funk et al. 2010). It has considerable psychological and social consequences for family carers (Wilkinson and Wilkinson 2020), especially if families decide to move care from home to hospice (Martz and Morse 2016). Furthermore, research on end‐of‐life care has tended to highlight the gendered nature of care, showing that although men are increasingly involved in caring for a dying spouse (Bertogg and Strauss 2020; Judd et al. 2019), the burden of caring for elderly relatives continues to fall to women (spouses, daughters and daughters‐in‐law) (Read and Wuest 2007).

Our focus in this article is on the relationship between care (at the end of life) and work, offering three key sociological contributions. Firstly, by unpacking how relatives juggle work and care in the temporally contested space of terminal illness, we extend the focus of sociological research on work and care. Secondly, by illuminating some of the medical but often hidden aspects of care, this article also contributes to sociological discussions on the notion of the carer as ‘health expert’. Finally, we analyse care through the lens of bereavement, thus complementing existing research on end‐of‐life care. By building an understanding of the *nature* and *meaning* of care across and beyond the boundaries of life and death, however, this article also offers an original contribution to broader sociological conceptualisations of care.

## 2 Narratives of Illness, Stories of Work and Care

Sociologists have illuminated how illness disrupts the taken‐for‐granted structure of everyday life (Bury 1988; G. Williams 1984). Such disruption can occur in temporal intervals (Harrison et al. 2024; McLaughlin et al. 2023) but often has long‐term physical, cultural, social, financial and medical implications for individuals and their families (S. Williams 2000). Episodes of illness often force people away from work, thus challenging their financial security and leading to a disruption in career trajectory and work identity (Watson 2009). A terminal diagnosis, however, presents individuals with a definitive disruption. Although lived experiences of illness are diverse, a terminal diagnosis sets a finite horizon and strips away an individual's future (Ehlers et al. 2024). Narrative reconstruction—the routine way in which we make sense of events in our lives, as sociologists have frequently shown—can provide individuals with an important way of imposing order on illness disruption (Good 1994; G. Williams 1984).

According to Bury (2001), language and narrative help sustain and create the fabric of everyday life, featuring prominently in the repair and restoring of meanings when they are threatened. It is no surprise, therefore, that narratives have provided medical sociologists with important conceptual and methodological tools that can be used to study illness experience and the social and cultural factors shaping that experience (Nettleton 2021). Narratives can perform many functions, from reconstructing an individual's life history in the event of illness to acting as a form of strategic interaction to assert or project individual identity (Hydén 1997, 155). Narratives also lack temporal continuity and are socially embedded and co‐constructed (Riessman 2015). This has been illuminated recently by the proliferation of personal accounts of the dying process on social media, which are often used to raise public awareness of different diseases (Brennan 2024). Both those experiencing illness and their significant others tell and retell illness stories (Kleinman 1988, 49), and it is through this process that illness moves from an individual to a collective experience (Hydén 1997).

This paper is inspired by a narrative of a health and illness approach but seeks to offer an original contribution in three respects: Firstly, the focus of much sociological research has been on the experience of the sufferer. We seek to enhance the analytical power of a narrative of illness framework by centring the illness experience but shifting the analytical focus from the ill person to their relative. Secondly, scholars of death and dying have shown how narratives from family members involved in end‐of‐life care can be used to examine the impact of illness on *their* social relationships (see Judd et al. 2019). We extend this focus by using accounts of illness as retold by bereaved relatives as a device through which to uncover the impact of illness on other aspects of *their* social lives—in this instance, care and work. Finally, although illness narratives frame much sociological work on chronic illness, terminal illness is often analysed through the analytical lens of death and dying. By using retrospective terminal illness stories as retold by bereaved individuals, this paper offers a novel empirical and substantive contribution to the sociology of health and illness.

## 3 Method

This paper is based on data collected from a 6‐month exploratory qualitative research project. The full project was conducted using two research methods: an open‐ended survey (*n* = 83) and follow‐up in‐depth interviews with 15 survey respondents. This article draws exclusively on interview data due to its scope and in‐depth focus. Interview participants were recruited via the survey once university ethical approval was secured. The survey was advertised by the researchers through Hospice UK newsletters and social media. The research title and all advertisements specifically used the words ‘looked after’ or ‘looking after’ someone who is dying, rather than ‘caring for’, to avoid alienating those who do not identify as carers. Survey data were used to build a picture of the background context for work and care. Participants were asked at the end of the survey if they would be willing to take part in an interview to leave their contact details. All those who left their contact details were invited to participate in an interview.

In total, 15 in‐depth interviews were conducted with 10 female participants and 5 male participants. At the time of interview, only 1 participant was actively involved in caring; the rest of the participants had cared previously until the point of death. Interview participants identified as White (European). They were engaged in a range of occupations (e.g., clerical, operations, accountancy and education). We recognise that there are limitations to our sample. For example, although some groups (such as women) were well represented in the research, others (such as men and individuals from certain minority ethnic groups and lower‐socioeconomic groups) were not. These limits relate to the short timeframe of the research and our inability to apply additional recruitment measures to target less well‐represented groups. We recognise that the responsibilities associated with care disproportionately fall on women and that other factors such as social class and ethnicity also inform the complex relationship between care and work. Because of the nature of our sample, we are unable to comment in detail on these issues in the context of this paper. The project on which this article is based, however, is an exploratory study that does not seek to offer a generalisable account of experiences of care and work at the end of life. Rather, it aims to offer insight into *some* of the challenges individuals face when attempting to navigate work while caring for a terminally ill relative.

**FIGURE 1:**
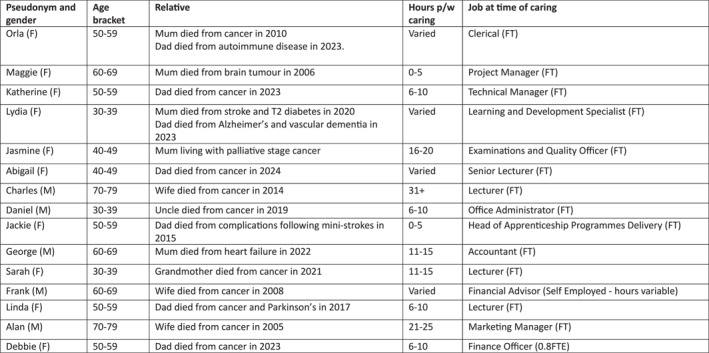
Participant information.

Participants in the interview part of the study all had different relationships to the people they were caring for, including partners, parents, aunts/uncles or grandparents. Figure 1 provides information on the nature of the care relationship and details the amount of time per week spent caring. Female participants were largely caring for parents, whereas men's involvement in different aspects of care suggests a complex and evolving picture (Bertogg and Strauss 2020; Judd et al. 2019; Read and Wuest 2007). The exploratory nature of this project means that we are unable to discuss these elements in detail. Rather, we aim to provide insight into the ways that people navigate work and care and suggest future research in this area.

Interviews were conducted both in person and online, according to the preference of the participant. We did not set out to study illness narratives or adopt the methodological approach of narrative interviewing. The narrative interview approach is a technique that eschews interview prompts, focusing instead on participant stories (Kartch 2017). Instead, we used an in‐depth interviewing approach (Mason 2017), whereby interviews were guided by questions informed by the survey data. For example, we asked participants to elaborate on the nature of their caring responsibilities, the support they received at work and how they navigated care with other life commitments (family, childcare) while caring. Although we did not explicitly adopt a narrative interview approach, participants used illness narratives (including a focus on diagnosis, prognosis and end of life) to detail their experiences of work and care. This methodological finding reinforces the argument that we are trying to make in this article more broadly about the importance and value of illness narratives beyond illness experience.

Once conducted, interviews were anonymised and transcribed. We took an inductive approach to thematic analysis, meaning that coding and theme development were directed by the content of the data. We began by reading and familiarising ourselves with the data, moving on to data coding, generating initial themes, reviewing those themes, defining and naming those themes, and then writing up (Braun and Clarke 2019). This was an iterative and reflexive process that took place throughout and beyond data collection. It became clear during the initial coding phase of the research that the concept of care was quite a problematic term for participants, and this tended to frame other themes. Such themes included the temporal nature of diagnosis and disease progression; managing work across life and death; relationality work and care; and structural barriers to work. In this paper, we focus on the relationship between illness, care and work, beginning in the first data section with the diagnostic journey.

## 4 Findings

In the following three sections, data will be presented through anonymised interview quotes. All participants and locations have been given pseudonyms. We start in the first data section by unpacking illness narratives, using these to begin to chart the relationship between illness and care.

## 5 Illness Narratives and the Meaning of Care

According to Hydén (1997: 55), illness narratives can perform many functions. They can act as a form of strategic interaction to assert or project a person's identity and transform the illness experience from an individual to a collective phenomenon. This section begins by exploring participant stories of their relative's diagnosis and illness journey. Through detailing these stories, we start to elucidate the nature and meaning of care participants provided. We also begin to uncover the biographical disruption that illness can cause to relatives, including their ability to work. In doing so, we illustrate how illness can impact not just the life and identity of the ill person but also those around them, thus further illuminating the socially embedded nature of illness.

Most participants began interviews by outlining their relative's illness—from initial diagnosis and treatment through to experiences of terminal illness and death. This is illustrated in the account from Frank, who was self‐employed and worked in financial services. He charts his wife's illness journey with breast cancer:

Cheryl, my wife. She was diagnosed with cancer in August of 2001, and went through treatment, went through a mastectomy. And then was coming up to the five years, which was July 2006, and then we were hit with the fact that it had spread to her other breast, and at that point, they said it was sort of palliative care. And from there onwards, it was basically downhill. She was put on a trial drug at Bart’s and for the first sort of three months, four months, that got rid of her back pain, she was mobile. And then all of a sudden, she started feeling very, very tired, there was no life in her whatsoever. The consultant at Bart’s said, basically the drug was wearing her out, it was a trial drug, it was wearing her out, so they took her off of it, and there was no going back after that, it was evident what was going to happen. And then in December 2008, that’s when she passed away.

As they recounted their relative's illness journey, participants began to illuminate the ways in which it disrupted their own lives. Through this process, they began to delineate the care they provided, outlining how this affected their paid work commitments. This is articulated by finance officer Debbie, who helped to care for her dad when he was diagnosed with terminal cancer. As her dad and stepmum lived in a different town over 20 miles from where Debbie lived, care required significant travel on a regular basis:

He (dad) and my stepmum lived in Tadbury. He got diagnosed last February with terminal cancer and he died last August, so it was, like, six months. I was the nearest, sort of, family member to be able to go and help out with things, so I was going there. I mean, I work Monday to Thursday, so I’ve got Fridays off, which was really great because I was going over there on Fridays. I don’t drive either, so it was lot of travelling back and forth to Tadbury. Going over there on Fridays and doing whatever they needed help with but also going to appointments and the hospital.

Participants tended to detail the practical tasks they performed for their relatives, frequently framing care in this context as an *activity* state (Thomas 1993). The type of care required, however, depended on the relationship of care. For example, partners often provided direct hands‐on care, whereas children might be caring indirectly through supporting one parent to care for the other. The nature of care was also contingent on illness type and disease progression. Lydia, for example, describes her mum's complicated health journey and reflects how that shaped the last few months of her life:

Then my mum, like I say, she’d had the stroke but she had many conditions, she’d got Type 2 Diabetes, her kidneys weren’t great because of that and we knew that. Whilst we’d been supporting my mum for a while and especially after my dad, in terms of just like shopping and seeing her and emotional support and things like that, it ended up that she got an infection and sepsis and her kidneys then failed. It was just that she was in hospital and they couldn’t make it better, they tried her on dialysis and it was just too much.

As sociologists have frequently shown, caring for someone with a serious or terminal illness can be a particularly intensive experience for family members (Thomas et al. 2002; Wilkinson and Wilkinson 2020). As with other forms of care (e.g., childcare, care of the infirm or elderly), it often involved participants engaging in a range of activities to improve the life of their loved one, including providing physical care and emotional support (Wilkinson and Wilkinson 2020). In our study, care frequently involved participants assisting their relative with medically related tasks—from changing a stoma bag to advocating with health professionals or administering pain‐relieving drugs. Such variety in care tasks is illuminated by Jackie, whose dad died from complications after a series of mini‐strokes in 2015.

It’s all the things that you just don’t think about to try and make him feel a bit better. Dressing. Covering my mum if she needed to go somewhere. Trying to advocate with medics. We went to A&E lots of times.

When engaged in providing medically related aspects of ‘care’, participants often appeared to take on the role of lay health expert. They learnt various medical skills—not through any official training initiatives—but from health professionals involved in their relative's care. This is illustrated by Katherine, who supported her dad to care for her mum and then went on to care for her dad when he developed bowel cancer. Katherine learnt how to change his stoma—a very intimate and physical form of medically related care. She felt that her dad preferred her to deliver such intimate care (rather than have a nurse do it), thus underscoring the value of informal family care in this context. She also discussed how she learnt medical techniques from specialist health professionals:

I mean, things like that don’t bother me, it’s just something, that’s what it was. I had to learn how to do it. I was taught by the stoma people.

Illness narratives, as G. Williams (1984) argued, can act as a reference point between the individual and society. In this section, we have shown how illness narratives can also act as a reference point for the relationship between *other* individuals and society. Illness stories retold by bereaved family members, as illustrated, can be used as an effective device through which to explore the disruption of illness on the lives of family members. Through detailing illness narratives, we have started to uncover the diverse nature of direct and indirect care administered in this context. Participant narratives also illuminate some of the more practical aspects of care, illustrating care as an activity state with intention and purpose (Thomas 1993; Reed et al. 2023). By showing how care in this context can go beyond physical and emotional tasks to include medical care and health advocacy, we have also begun to extend sociological understandings of care in this context. We move on in the following section to focus more directly on the relationship between illness, care and work.

## 6 Illness Disruption and the Challenge of Work

Sociologists have frequently shown how illness disrupts the taken‐for‐granted structure of everyday life (Bury 1988; G. Williams 1984) and can force people to reflect on work identity and do identity work (Watson 2009). Research on informal care has also shown how paid work is often incompatible with long‐term caring responsibilities (Barrett et al. 2014; Bernard and Phillips 2007; Geisen et al. 2024). In our research, the onset and progression of their relative's terminal illness significantly disrupted participants' lives. It could, at times, however, also provide them with space to reflect on their own lives, work identity and priorities. In this section, we explore how participants navigate work and care commitments in the ambiguous and uncertain context of terminal illness and end‐of‐life care (Rovers et al. 2019), noting the various challenges that they face. Through this process, we will also begin to illuminate the ways in which biographical disruption, brought on by their relative's illness, could lead participants to re‐evaluate their own priorities.

All participants felt they were constantly juggling the demands of work and care. This often involved being available to relatives to provide physical care and emotional support, as well as aiding with navigating healthcare systems. For example, participants often had to take time off work to take relatives to medical appointments. What was clear in participant accounts, however, was the ways in which the health of their relative often took priority over work commitments. This is articulated in the extract from Frank, who was self‐employed and had some flexibility over the organisation of work. He juggled various work commitments with his wife's hospital appointments during her treatment for cancer but stated that hospital appointments always took precedence:

And that’s what I was doing, you know, you fitted it in, if Chery had an appointment at nine o’clock, I’d make sure she was there, and I’d adjust my work around that time.

Research has highlighted some of the challenges individuals face when trying to juggle work and caregiving responsibilities, emphasising both the emotional and physical burdens and pressures combining the two roles can place on individuals (Jimenez et al. 2019; Lehner et al. 2012). This is something that was articulated by all our participants—regardless of the care relationship (whether child, partner or nephew). For example, as Charles, whose wife died of cancer in 2014, conveys: *‘I was feeling under pressure to get here (work) and do what I had to do and then get back (home) again. So it was, yeah, I think a negative experience on balance’*.

When it came to organisational culture and work environment, participants' views were often mixed. Most participants felt like their line managers and colleagues were supportive, with several participants detailing how flexible their line manager was. This is articulated by Sarah, whose grandmother had died of cancer in 2021:

It was informal, flexible. My manager was really supportive of that: so as I say, I never went off sick, but she was also like, we’ll do what we can if we need to we’ll go down that (formal) route, but we’ll just see how it goes, you just keep me updated. So it was never her chasing me to go, what’s going on, how was she this week, how do you think you’re going to do this week; it was always more upon my terms that I could update her and kind of approach her when I needed to.

Not all participants had colleagues and line managers who were supportive, however, as illustrated by Linda's experience. Linda, whose dad died from cancer and Parkinson's disease in 2017, did not live in the same geographical region as her dad. As his illness became more advanced, Linda's dad required greater formal multiagency intervention. This meant that, alongside visiting him more frequently, she also spent significant time on the phone to health and social care providers, trying to arrange formal care. She felt that her work colleagues were very intolerant of her situation:

And yeah, I can remember someone in the corridor, just slamming their door, and thinking, oh, okay, yeah. And then maybe I’d be at work, and I’d get a phone call from the social workers. So they were really hard calls not to take, when I’m at work. And then I was asking for leave, ‘cause I’d arranged to see a social worker and I’d drive all the way down and the social worker would be on annual leave or something. It was just, oh, so stressful.

Many participants took advantage of the reorganisation of work practices and the shift towards hybrid working, which has become more commonplace since the onset of COVID‐19 (Reed 2024). The ability to work at home combined with having a supportive line manager made a huge difference. This enabled participants to accompany their relatives to hospital appointments for tests and treatment as articulated by Katherine in the quote below:

So, he (line manager) always knew and he always said to me, look, if you need to be off, don’t tell me, just drop me an email. You don’t have to ask, just don’t come in. And there were a couple of days when he’d got appointments at home and things or I had to take him to chemo or there was something that he had to go to the hospital for bloods or whatever, that I just said, can I work from home tomorrow?

Although most participants attempted to juggle work with care responsibilities, when a diagnosis was terminal, participants' work often took on a less important role in their lives. This is articulated by Daniel, whose uncle died from cancer in 2019: *‘Well, my life really just took a bit of a back seat. I didn't really do much. Didn't go for any promotions, didn't meet anyone’*. His uncle's illness disrupted his life and ability to work, causing him to reflect more broadly on his work identity. This disruption was not a wholly negative experience for Daniel, however. The disruption caused by the illness along with the care his uncle required brought his family closer together:

My job, I was grade four (administrative role), I’ve not got loads of responsibility on my shoulders, I just sit there and answer emails all day, so me doing that at home. My line manager was always great with me. At that time, as I say, what was going on with my uncle, it was nice to be there, he brought the family together a bit more, my brother and dad eventually came over. Because mine and my dad’s relationship is loads better off all the back of this because we got to spend some time that wasn’t just arguing and stuff like that together.

Working while supporting a dying relative could, as shown in this section, be very challenging. Care often involved taking relatives to hospital for tests or treatment, sometimes travelling significant distances to do so. Participants' ability to do this was often contingent on them having a supportive line manager and workplace flexibility (Geisen et al. 2024). As Daniel's account showed, however, the disruption caused by their relative's illness did lead participants to reflect on their work identity with development, career and promotion taking a back seat (Watson 2009). This reinforces the power and value of using illness narratives as a tool to examine the profound effects of illness on social lives and worlds beyond those of the sufferer. The more participants talked about their relative's disease progression, care and work, the clearer it became in their accounts that family, rather than work, was their priority. This commitment was made possible by the fact that terminal illness is time‐bound, and it is less possible to prioritise family over work when caring responsibilities are ongoing. This is something that will become more apparent as we examine time, work and care in the context of disease progression in the final data section.

## 7 Time to Care? Identity, Work and Disease Progression

The terminal phase of an illness signals a disruption to both the pace and dominance of time (Rovers et al. 2019). The contours of time running out structure the everyday lives of individuals with a terminal diagnosis as well as those of their families (Broom et al. 2016; Broom et al. 2019, 2020; Ehlers et al. 2024). Past, present and future conceptualisations of time become ambiguous in the face of end‐of‐life uncertainty (Ehlers et al. 2024; Rovers et al. 2019). As Davies (1994) has illustrated in her research on childcare, care is a temporally located activity which involves multitasking across time and place but is always enmeshed in social relations (Davies 1994). In this section, we explore time through participant narration of the final stages of their relative's illness, uncovering the growing intensity of care during this time. Through this process, we illuminate the uncertainty of time along with the ways in which time is embedded in social relationships.

Our interviews showed how the care needs of relatives often became more acute over time as their illness progressed. This had significant implications for our participants, who were required to become more involved in direct care as well as advocating for (health) care. Participants often conducted multiple care activities across time and place (Davies 1994). This is articulated by Linda, whose dad's care needs became more acute towards the end of his life. She had to take time off work to liaise with social workers directly to sort out her dad's care:

So he was back in the acute hospital. So every incident like that, he reduced and reduced, so yeah. So then I remember taking some more annual leave and going down and being told by the social workers, if you don’t find anywhere for him by the end of today, we’ll find a care home for him, and the nearest one we know of is in Gloucester

Participants in our study frequently spoke about the uncertainty of death, anticipating what those final days would be like. Research has shown that this is common among those experiencing terminal illness (Cleeve et al. 2018). In such instances, participants often tried to educate themselves about the illness in question to gain some temporal certainty. For example, George spent lots of time ‘reading up and learning about heart disease’ through the British Heart Foundation to understand his mum's prognosis but ultimately felt left in the dark about how things might unfold because ‘there is no accurate scale for heart failure’.

Such uncertainty had significant implications for how our participants managed their lives. They could be told by medical professionals that their family member was close to death only to find that they hung on in an interstitial space between life and death for months or even years. This is articulated by Maggie below, regarding her mum who died of a brain tumour in 2006:

We didn’t know how long she was going to last, because there was a point in August 2005, when we…we were given the indication, sort of vaguely, that she’d gone downhill so badly that she went to a hospice for a couple of weeks, and we didn’t think at that point that she was going to come back out again. But she did, and she lasted then for another, almost another year at home.

Our data reinforce the ways in which time is embedded in social relations (Davies 1994; Riessman 2015). Most participants wanted to spend as much time as they could with their relative when they were close to death but were often unable to take extended periods of annual leave to do this. Participants often completed work tasks at night to keep up with work so they could spend more time with their relative during the daytime. Their ability to do this was often contingent on their employer being flexible and compassionate, as illustrated by Katherine:

So, I just did everything on an evening. Appointments my boss allowed me to go to. And then until it got really, really bad to the stage like right at the end, I just said, look, I’m not coming into work because he needs me, I’ve got to stop at home with him and it doesn’t look like he’s going to last less than a week anyway. And my boss was like, yeah, no problem. If I needed more time off, he would have given me more time. But I didn’t, I did it on an evening.

Through detailing her own illness experience of cancer diagnosis and treatment, Riessman (2015) makes the important point that illness narratives do not follow linear conceptualisations of time but are in fact disordered and temporally ambiguous. This was certainly the case in our study. As they detailed the final stages of their relative's illness, participants tended to reflect on the original diagnosis and treatment, sometimes articulating regrets. Although participants did all they could to prioritise spending time with their ill relative, they sometimes wished that they had spent more time with them in the earlier stages of their illness. This is articulated in the quote from Charles: ‘*I think if we'd known it was going to be so short, I would have probably done everything differently from how I did’*.

Several other participants articulated similar feelings of guilt. They felt that they had struggled to navigate work while also wanting to spend as much time as possible with their ill relative. Such feelings are articulated by Alan. His job was at risk during his wife's initial cancer diagnosis. He felt at the time that his focus had been on keeping his job, and he worried that this had impacted negatively on his wife's prognosis.

Well at the time, we'd not got that prognosis. Looking back, I wish I'd been more on the ball. I don't think there's anything we could have done that would have averted her death. But I think there's stuff we could have done that would have prolonged her life. But at the time I was just going in and supporting her in hospital visits. Eventually she was diagnosed because she had this bone scan that uses isotopes, a full body scan. It was the first of the summer barbecues that she complained of back pain, so it would be late August, so that was four months. I’m not blaming myself, but my focus is on keeping my job.

Despite the challenges and exhaustion, however, participants all valued the time that they had spent with their relatives. It was not uncommon for participants to talk about how they cherished these moments together. Care in these final stages of life, therefore, was often not viewed as a form of labour but as an act of love. This is articulated by Sarah in the quote below:

So actually, it was really nice as a way of kind of, we’ve got these moments together. And I know my grandma didn’t want to feel like a burden; there was a lot of time she felt like a burden, and she was like, I feel really bad that you’re here and you’re having to care for me. But we were like, we want to. And I think that makes a big difference, that it wasn’t a case of we have to, it was a case of we wanted to; so that’s probably why it felt less like a second job.

This section has illuminated how participants' engagements in care change as the end of life becomes nearer. Time in this context sped up as death loomed on the horizon, making the navigation of work and care more difficult. As Ehlers et al. (2024) point out, terminal status might mark the reality that death is imminent, but this reality is lived in complicated ways. This was certainly the case in our study as participants tried to navigate an ever more complex and temporally uncertain terrain. Talking about their relative's end of life made participants more reflective about their illness, thus reinforcing the temporal ambiguity of illness narratives themselves (Riessman 2015). As end of life approached, however, work appeared to take a backseat in participants’ lives because they wanted to spend as much time as possible with their relative. This shows how time and care are enmeshed in social relationships (Davies 1994). It also illuminates how the relationship between care as an *activity state* and care as a *feeling* is heightened as death approaches (Thomas 1993). This is something we will reflect upon further in the conclusion.

## 8 Discussion

This article has examined the biographical disruption that terminal illness can cause, not in this instance to the life of the sufferer, but rather to those around them. Although terminal illness might impose a finite horizon, it is not, as shown through our participant narratives, a linear experience (Ehlers et al. 2024) but rather involves a process of ongoing reflection (Riessman 2015). In this article, we have shown how terminal illness can prompt relatives into doing *identity work*, including reflecting on their own *work identity* (Watson 2009). Through detailing their relative's illness journey, participants constructed their own identities around work, care and family. This reinforces how illness and illness narratives are deeply socially embedded (Brennan 2024; Riessman 2015). It also shows how illness narratives can provide an important sociological tool which enables us to analyse other *social lives* and *social worlds* beyond direct illness experience. This is something, perhaps, that sociologists using narratives to examine other aspects of social life could consider. In this article, we have unhooked the illness narrative approach from the experience of the individual sufferer, using illness as a proxy through which to analyse the relationship between work and care. In doing so, we have sought to highlight the value of using the illness narrative approach beyond its current sociological moorings.

We have also sought to contribute to wider sociological literature on care by shedding light on the nature and meaning of informal care in the context of terminal illness. Care in the context of terminal illness often includes medically focused tasks (e.g., advocating for care and administration of medical care). These tasks, we argue, could be viewed as family members becoming a lay ‘health expert’. Such expert knowledge, we argue, is not an extension of state surveillance as was the case in Sadler et al. (2018) study on informal care in stroke units. This is because our participants did not participate in formal training programmes but rather acquired medical knowledge through reading, learning and engaging with health professionals about medical tasks. There are, of course, limits to lay medical knowledge. As Prior (2003) notes, laypeople have experience which—although valuable—does not equate to the technical knowledge required to be granted the status of expert. Acknowledging the limits of the term lay expert, however, we want to use it here to move sociological debates on care in the context of terminal illness beyond emotional and physical labour. Although informal care of the terminally ill family member *is* emotionally and physically demanding, new knowledge may be acquired through this form of care, provoking the construction, perhaps, of new identities of care. This indicates a need for sociologists to consider broadening their understanding of what constitutes care in different social contexts.

Care, as we have also sought to show throughout this article, varies according to illness type and status as well as the social relationships involved. Care, as we have shown, also varies according to time and context (Davies 1994). Qualitative data from our research illustrate how the relationship between care as *activity state* and care as *feeling* becomes heightened as death approaches, with care as *feeling* taking centre stage. This illustrates the importance of acknowledging not only the temporal nature of care and the temporal ambiguity of terminal illness but also, perhaps, how care at the end of life can manifest as the ultimate act of love (Cataldi 2023; Pulcini 2013). According to Tronto (2015), care is about meeting needs, and such needs end with death. But although death may signal the end of care as an *activity state*, reflective narrative accounts from our participants show how care as *feeling* continues beyond death. This reinforces the need for sociologists to take a more nuanced approach to care, recognising the ways in which the categories of care change according to time and social context, across and beyond life and death.

Finally, although we have sought to scrutinise the concept of care in this article, our focus has been on analysing the impact of terminal illness and care on work. Our qualitative data reinforce the findings of existing empirical research, noting how difficult it can be to balance informal care and work effectively (Geisen et al. 2024). The opportunity for hybrid working, combined with having a supportive line manager and colleagues, made a huge difference to our participants. Both factors could help participants to manage the pressure of trying to combine work and care. This was especially important for those individuals who were geographically commuting to care. Existing research shows how workplace bereavement support policies are often very limited or even nonexistent, their effective actualisation often contingent on having a flexible and supportive line manager (Reed 2024). An employee's ability to effectively care for a relative when they are dying, as our data illustrate, is also contingent on the flexibility and adaptability of a compassionate line manager. This is an area of work, therefore, where more policy‐focused research is needed.

## 9 Conclusion

This article has explored experiences of caring for a relative who is terminally ill and the impact this can have on an individual's ability to work. By drawing on the narrative of health and illness approach and illuminating some of the more medical aspects of care, we have sought to anchor our analysis squarely within the sociology of health and illness. Sociologists have warned against over‐privileging the role of narratives in human lives, creating what Phelan (2005) refers to as ‘narrative imperialism’ (210). It is important not to overemphasise the value of narratives, but, as Watson (2009: 429) points out, they do play their part in the lives of all of us, *regardless of whether we are particularly self‐conscious about it*. This is illustrated in our study, which did not aim originally to adopt a narrative of health and illness approach but which did end up collecting powerful and compelling illness stories. Furthermore, as sociologists of health and illness have long argued, illness narratives can tell us so much about the relationship between individuals and society and how this can shape experiences of illness (Nettleton 2021). They can also illuminate other aspects of social life and, as we have sought to advocate here, can be used to examine other social phenomena beyond illness itself.

There are limitations to what we have discussed in this article. For example, the small‐scale nature of the study and diversity of relationships involved make it hard to generalise. Furthermore, although one of our participants was self‐employed, the rest were employed in permanent posts. We cannot comment, therefore, on the experiences of those engaged in more precarious forms of employment. It would be beneficial, therefore, for future sociological research to expand this to examine the impact of illness and care on individuals engaged in a broader range of occupations—including those perhaps engaged in precarious forms of employment (gig economy and 0‐hour contracts). This would enable future sociological analyses to unpack the complex socioeconomic relationship between work and care at the end of life. It would also be beneficial for further research to examine other types of care relationships, to really capture the demands of caring in that interstitial space between life and death.

## Author Contributions

**Kate Reed:** conceptualization, writing – review and editing, methodology, writing – original draft, formal analysis, investigation, funding acquisition. **Laura Towers:** investigation, formal analysis, methodology, writing – review and editing, project administration, data curation, funding acquisition.

## Funding

It was conducted using discretionary funds from the University of Sheffield where both authors worked previously.

## Ethics Statement

This project received ethical approval from the University of Sheffield.

## Consent

All research participants provided informal consent.

## Conflicts of Interest

The authors declare no conflicts of interest.

## Permission to Reproduce Materials From Other Sources

All material in this paper is original and belongs to the authors.

## Data Availability

The data that support the findings of this study are available from the corresponding author upon reasonable request.
